# Acute exposure to hyperthermic oscillating temperatures during pre-incubation influences northern bobwhite development, hatching, and survival

**DOI:** 10.1371/journal.pone.0219368

**Published:** 2019-07-10

**Authors:** Kelly S. Reyna

**Affiliations:** College of Agricultural Sciences and Natural Resources, Texas A&M University – Commerce, Commerce, Texas, United States of America; Tokat Gaziosmanpasa University, TURKEY

## Abstract

Temperature extremes alter development, growth, hatching, and survival of eggs of ground-nesting birds, particularly during pre-incubation (egg laying) when eggs are left unattended and exposed to the environment for days or weeks before parental incubation begins. The northern bobwhite quail is a ground-nesting bird whose eggs experience high temperatures (≥45° C) during pre-incubation. It is known that chronic high temperatures during pre-incubation alter development and reduce hatching and survival of bobwhite eggs, but it is not known if acute doses of high temperatures during pre-incubation have the same effect. In this study, the 12-d pre-incubation period was divided into thirds. Fresh bobwhite eggs were exposed to either a commercial holding temperature for all 12 d (serving as a control), or a high oscillating temperature regimen for 4 d (one third of pre-incubation) either in the early, middle, or late third of pre-incubation, with a low oscillating temperature regimen during the remaining 8 d. The timing of acute exposure to high oscillating temperatures significantly affected bobwhite development. Eggs exposed in the first 2/3 of pre-incubation developed twice as much as eggs exposed late in pre-incubation, even though all eggs received the same amount of heating degree-hours. Thus, a critical window of thermal susceptibility exists for developing northern bobwhites. Acute exposure to high oscillating temperatures resulted in reduced hatchling mass, hatching success, survival, and compromised hatching synchrony. Thus, acute hyperthermic nest temperatures during pre-incubation could result in the observed reductions in the percentage of juveniles in natural populations during hot and droughty years.

## Introduction

One of the most important environmental forces acting on avian embryos is temperature which drives embryonic development, growth, hatching, and survival [1–5]. The study of the interdependence of temperature and avian ontogeny has largely been directed towards determining the optimum incubation temperature to achieve the highest growth and hatchability for commercial production [1, 6–15]. Thus, many studies assessing the impacts of temperature on avian embryos use constant incubation temperatures equal to the commercial standards of ~35–38° C [1, 10–12], or constant temperatures equal to the mean daily temperatures of a species’ natural environment [16, 17].

However, the embryos of many precocial birds who lay multiple eggs in a clutch experience an oscillating thermal environment during pre-incubation; as compared to the relatively constant temperatures experienced during incubation [4]. The hens deposit eggs in their nest at a rate of one egg per day and do not begin incubation until the penultimate or ultimate egg is laid [2, 18]. Thus, there is a pre-incubation period where eggs are unattended and exposed to the diurnal thermal oscillations of the environment, prior to incubation [19]. Oscillating temperatures during pre-incubation have been shown to have much different effects on the development, growth, hatching, and survival of avian embryos when compared to constant temperatures, even with the same amount of heating degree-hours [4].

The temperatures of the diurnal oscillations during pre-incubation, for species like northern bobwhites (*Colinus virginianus*), are often extreme and even lethal in southern latitudes [5]; regularly exceeding 40° C, peaking at 45° C in drought years, and even reaching 60° C [4, 5, 12, 19, 20]. Simulating these extreme pre-incubation oscillating temperatures in the laboratory has been shown to decrease northern bobwhite egg hatching rates by ½ compared to simulated normal, or non-drought conditions [4]. These results are very important to understanding the range-wide decline in bobwhite populations.

To better understand the effects of diurnal temperature fluctuations during pre-incubation, we need to know more about the impacts of the timing and duration of exposure to the stressor [21]. Temperature perturbations have been shown to elicit developmental plasticity in vertebrates depending on the timing of exposure [1, 21–23]. Such time or age dependent results suggest that critical windows of development exist [1, 21–24] in which an embryo is most susceptible to temperature stressors. Thus, it is plausible that bobwhite embryos exposed to equal durations of oscillating hyperthermic temperatures at different time periods within pre-incubation (early, middle, and late stages of pre-incubation, for example) will express plasticity of development, hatching, or survival.

Accordingly, the objectives of this study were to test for critical windows of northern bobwhite development during the pre-incubation period to determine whether a time or age dependent response to diurnally oscillating hyperthermic temperatures exists. For example, would eggs exposed to high temperatures at the end of the pre-incubation period develop, hatch, and survive better than eggs exposed to high temperatures during the beginning of pre-incubation? It was hypothesized that exposing bobwhite embryos, *in ovo*, to equal heating degree-hours of oscillating hyperthermic temperatures, administered in different periods of the pre-incubation period (early, middle, and late), would cause plasticity of development, hatching, and survival. That is, with equal doses of heat administered at different times in pre-incubation, the results of development, hatching, and survival would be different. This study is important because northern bobwhites are listed as a near threatened species by the International Union for Conservation of Nature. Populations in their semi-arid range strongly correlate with drought, a time when the species encounters hyperthermic heat loads [19, 25–27]. Thus, determining if acute doses of high heat impact developing northern bobwhites as much as chronic doses [4], will better inform future management decisions and improve conservation efforts.

## Materials and methods

Fertilized northern bobwhite eggs were collected from captive-reared breeding pairs of flight ready birds at Lake Cumberland Game Bird Farm (Mill Springs, KY, USA). Eggs were packaged and shipped to the laboratory on the day of collection. Eggs arrived on-site within 48 h with a written record of the date and time of egg collection. 3 shipments of 68 eggs were made. For each shipment, bobwhite eggs were randomly divided into 4 groups (3 treatment, 1 control) of 17 eggs, placed on plastic egg trays blunt end up, and given a unique identifying number with an indelible marker. Each egg was then weighed to the nearest 0.01 g with a digital scale (Ohaus Explorer Pro, Pinebrook, NJ, USA) and placed into the assigned pre-incubation treatment and experiment described below. The experiment was conducted 3 times for a total of 60 eggs per group.

Lake Cumberland Game Bird Farm was approved for egg production by the United States Department of Agriculture (USDA) and certified by the USDA National Poultry Improvement Plan. This research was approved by the University of North Texas Institutional Animal Use Care Committee, protocol # 0808.

### Pre-incubation treatment

Egg groups were assigned to a thermal chamber (G.Q.F. 1583 Hova Bator with circulated air; RH = 60%) with a unique experimental thermal regimen based on the timing of exposure to doses of low oscillating temperature (LT), high oscillating temperature (HT), or commercial temperature (CT) during the 12-d pre-incubation period (Table 1). The peak temperatures of the low and high groups were selected based on nesting studies showing temperatures peaked ≥40° C in non-drought years and ≥45° C in drought years [4, 19, 20, 25]. The nature of each treatment was such that the LT treatment exposed eggs to 92.4 heating degree-hours per day, the HT treatment exposed eggs to 212.4 heating degree-hours per day, and the CT treatment received no heating degree-hours during pre-incubation [4]. A heating degree-hour was defined as 1° C above physiological zero for 1 hour where physiological zero is 25° C for northern bobwhites [28]. Relative humidity (RH) was maintained at 60% in all thermal chambers and eggs were not turned during pre-incubation.

**Table 1:** Diel thermal schedule of pre-incubation thermal treatment categories.

Time of Day	Low Temp (LT)	High Temp (HT)	Commercial Temp (CT)
0000–0759	25° C	30° C	20° C
0800–1059	30° C	35° C	20° C
1100–1359	35° C	40° C	20° C
1400–1659	40° C	45° C	20° C
1700–2359	25° C	30° C	20° C

Treatment egg groups were exposed to 4 d (one third) of the HT diel thermal regimen either in the early (HT_Early_), middle (HT_Middle_), or late (HT_Late_) third of the 12-d pre-incubation period, with the remaining 8 d of the pre-incubation period in the LT diel thermal regimen (Table 2). All HT treatments received 1588.8 heating degree-hours during pre-incubation. The fourth group (CT) was exposed to the commercial temperature for holding eggs (20°C) for the duration of the pre-incubation period and received no heating degree-hours.

**Table 2:** Pre-incubation (PI) schedule used to expose northern bobwhite eggs to acute doses of hyperthermic heat either in the early, middle, or late PI period.

Group	12-d Pre-incubation (PI)		
	PI days 1–4	PI days 5–8	PI days 9–12
High Temp Early (HT_Early_)	**HT**	LT	LT
High Temp Middle (HT_Middle_)	LT	**HT**	LT
High Temp Late (HT_Late_)	LT	LT	**HT**
Commercial Temp (CT)	CT	CT	CT

### Incubation

After the 12-d pre-incubation treatment was complete, eggs from each group were removed from the thermal chamber, weighed to the nearest 0.01 g, and immediately placed into an incubator (G.Q.F. 1502 Sportsman incubator, G.Q.F. Manufacturing Co., Savannah, GA, USA) to begin a standard incubation [4, 29]. Incubator temperature was maintained at a constant 37.5 ± 0.5° C with RH = 60%. Eggs were turned automatically every 3 h for the first 19 d of the 23-d incubation [1]. On day 20 of incubation, eggs were removed from the incubator, weighed to the nearest 0.01 g, and placed in the hatching chamber of the same incubator, with no egg turning [1].

### Analysis of development

After the 12-d pre-incubation treatment, on the 13^th^ day of the experiment, 7 eggs from each group were randomly selected for developmental staging [4, 30, 31]. Selected eggs were weighed to the nearest 0.01 g, to determine the amount of water loss during pre-incubation, and subsequently opened. If embryos were present, they were separated from the egg (yolk-free), weighed to the nearest 0.01 g, aged and staged according to morphological indicators of development [4, 30, 31]. The remaining 15 eggs per group were placed in incubation.

During incubation, on incubation days 10, 13, 15, 17–22, and 1-d post hatch, Vo2 (oxygen consumption) was recorded for 2–6 randomly selected eggs or hatchlings from each group via flow-through respirometry. Oxygen consumption was used as an indicator of development (more developed embryos consume more oxygen) and to determine the timing of internal pipping (when embryos begin air breathing in the egg) as described previously by Reyna and Burggren [4]. Randomly selected eggs from an individual group were removed from the incubator and immediately placed into individual metabolic chambers located in a modified incubator as part of the flow-through respirometry system [4]. The chambers and influent air were maintained at normal incubation temperature (37.5 ± 0.5° C) so that no heat loss occurred during testing. Each chamber was sampled at 5 sec consecutive intervals for 15 min. After Vo2 recordings, eggs were immediately placed back into the incubator to minimize heat loss. A subset of incubating eggs (10 eggs per group total) was allowed to complete the hatching process and were subsequently maintained for one day at 37.5 ± 0.5° C with a RH of 60% for hatchling oxygen consumption measurements.

### Analysis of hatching

For this study, eggs were visually observed daily. The timing of internal pipping was recorded when the embryo pipped into the egg air as determined visually when candling. Internal pipping was also indicated by a sudden increase in Vo2 in the flow-through respirometry system. Thus, Vo2 measurements were taken on incubation days 17–24 to ensure the increase was observed. External pipping was recorded when the embryo first pipped through the eggshell. Hatching was recorded when eggs were star-pipped—when an embryo creates a small hole in the shell to initiate hatching [4, 32]. Once star-pipped, eggs were placed in a glass desiccator with isoflurane, embryos were subsequently removed from the egg and indicators of development, including wet mass, 3^rd^ toe length, and beak length were recorded [4, 30, 31]. Hatching success was recorded as the percentage of eggs hatched (star-pipped) per treatment group.

### Analysis of survival

After pre-incubation and on days 20–24 of incubation, eggs that where not star-pipped were candled to assess viability, as indicated by observed movement or size. The time to mortality as indicated by the developmental stage at the time of death was recorded for any egg that was not viable [30, 31].

### Statistical analyses

All data were tested with a Shapiro–Wilks normality test and Hartley’s F_max_ test before specific statistical analyses are performed [33]. A one-way ANOVA was used to identify the relationship between groups for all experiments. A Kaplan-Meier survival analysis [34] was used to compare survival rates and time to mortality among groups. Significance between groups was determined with a Student–Newman–Keuls (SNK) multiple range *post hoc* test [33]. All statistical tests were conducted using SigmaPlot 14.0 software (Systat Software Inc. San Jose, CA). Statistical decisions were made with a 0.05 level of probability. All data are reported as mean ± S.E. unless otherwise indicated.

## Results

### Pre-incubation development

**Fig 1:**
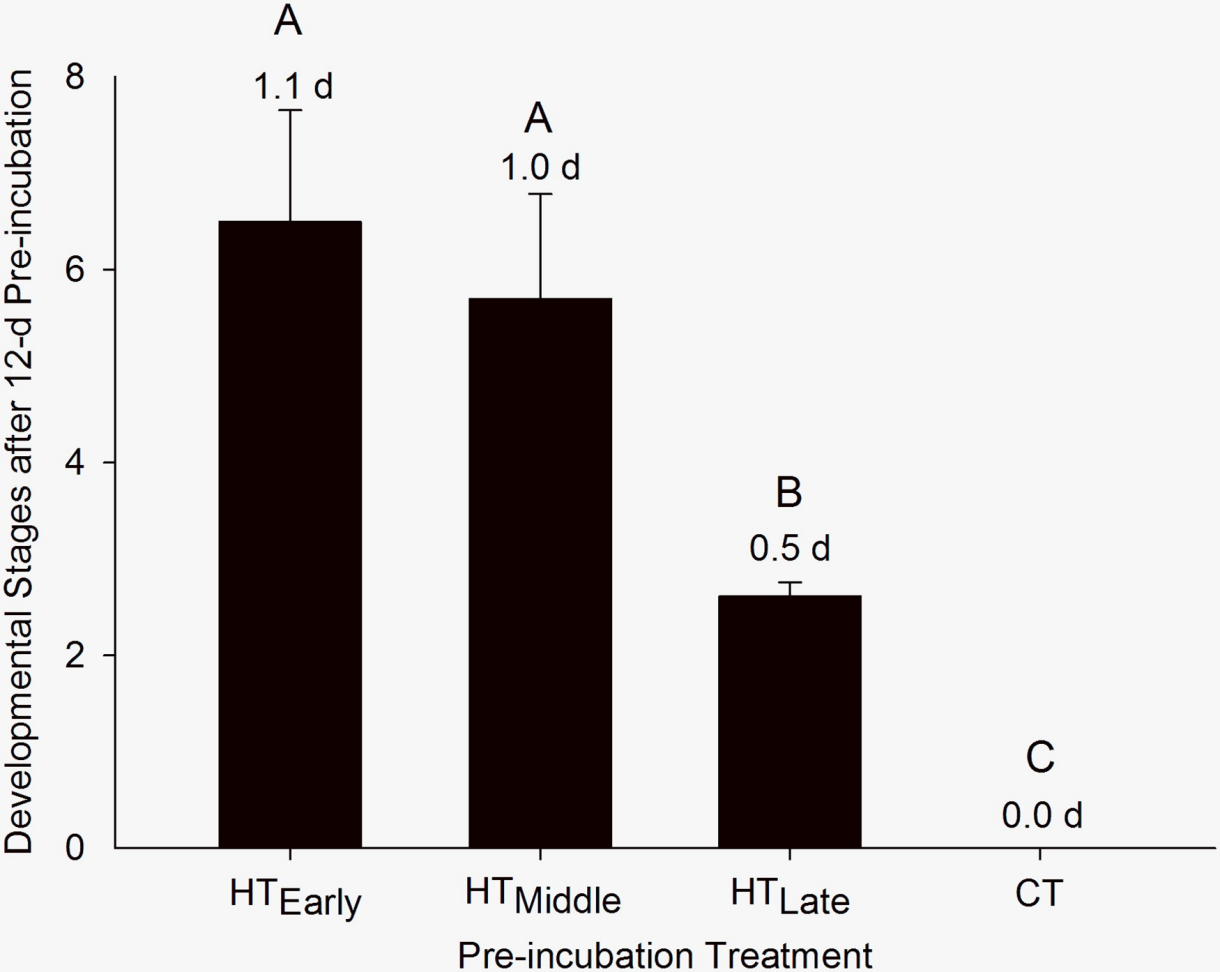
Mean developmental stages of northern bobwhite embryos exposed to high temperatures (HT) in the early, middle, or late third of a 12-d pre-incubation period. Numbers above bars indicate days of incubation equivalent to stage (i.e., stage 6.5 occurs at 1.1 d of commercial incubation). Letters indicate statistical groupings. Data are presented as mean ± S.E., N = 21 (HTEarly, HTMiddle, CT), and 19 (HTLate).

Eggs exposed to acute doses of HT during pre-incubation displayed differential development even though each group received 1,588.8 heating degree-hours (Fig 1). Eggs in the HT_Early_ and HT_Middle_ groups were more responsive to the hyperthermic conditions, advancing to stages 6.5 ± 1.1 (equivalent to 1.1 incubation days) and 5.7 ± 1.0 (equivalent to 1.0 d of incubation), respectively. HT_Late_ eggs, receiving HT exposure in the last 3^rd^ of pre-incubation, were less responsive to thermal stress and only advanced to stage 2.6 ± 0.1, the equivalent of 0.5 d of incubation at a constant 37.5° C (Fig 1). CT eggs received no heating degree-hours during pre-incubation and did not begin developing during this time (Fig 1).

### Egg mass and water loss

**Fig 2:**
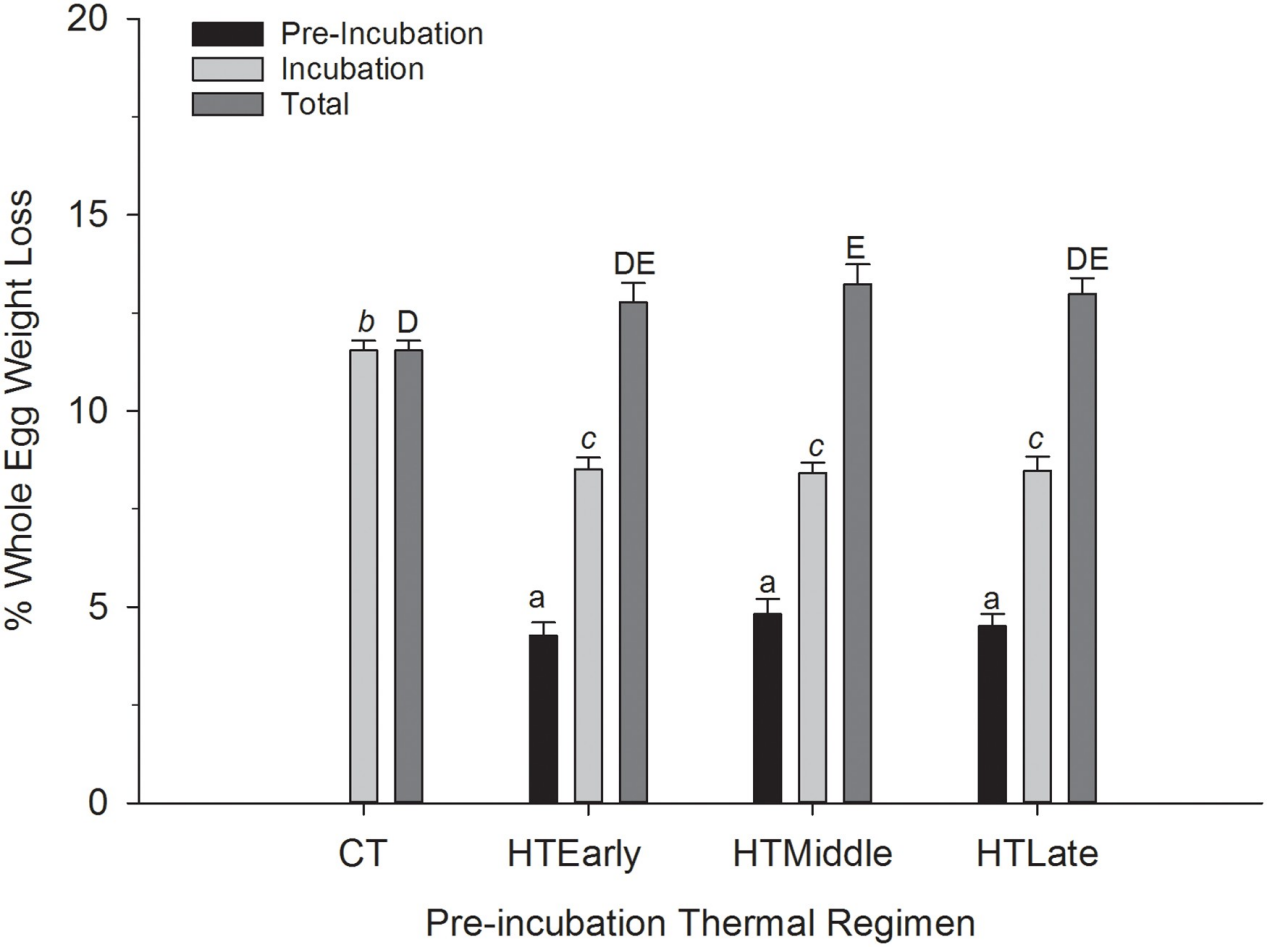
Mean percentage of fresh egg weight loss for northern bobwhite eggs exposed to high temperatures (HT) in the early, middle, or late third of a 12-d pre-incubation period. N = 10 (HTEarly), 19 (HTMiddle), 14 (HTLate), and 19 (CT). Letters indicate statistical groupings with a 0.05 level of significance.

The difference in mean percent fresh egg weight loss during pre-incubation among HT groups was not significantly different (P = 0.22). HT_Early_ eggs lost 4.2% ± 0.3 of fresh egg mass during pre-incubation. HT_Middle_ eggs lost 4.8% ± 0.3, and HT_Late_ eggs lost 4.5% ± 0.3. CT eggs did not lose any mass during pre-incubation (Fig 2).

During incubation, HT egg groups lost less fresh egg mass than CT eggs (P<0.001). CT eggs lost 11.5% ± 0.2 of fresh egg mass. HT_Early_, HT_Middle_ and HT_Late_ egg groups lost similar percentages of fresh egg mass, 8.5% ± 0.2, 8.4% ± 0.2, and 8.4% ± 0.3 respectively (P = 0.99; Fig 2).

Combining the pre-incubation and incubation periods for total weight loss, HT_Middle_ eggs (13.2% ± 0.5) lost a greater percentage of fresh egg mass than CT eggs (11.5% ± 0.2; P = 0.04). However, there was no difference between the mean percentage of total weight loss between HT groups or between CT and HT_Early_ (12.7% ± 0.4), and HT_Late_ eggs (12.9% ± 0.4; Fig 2).

### Incubation development

**Fig 3:**
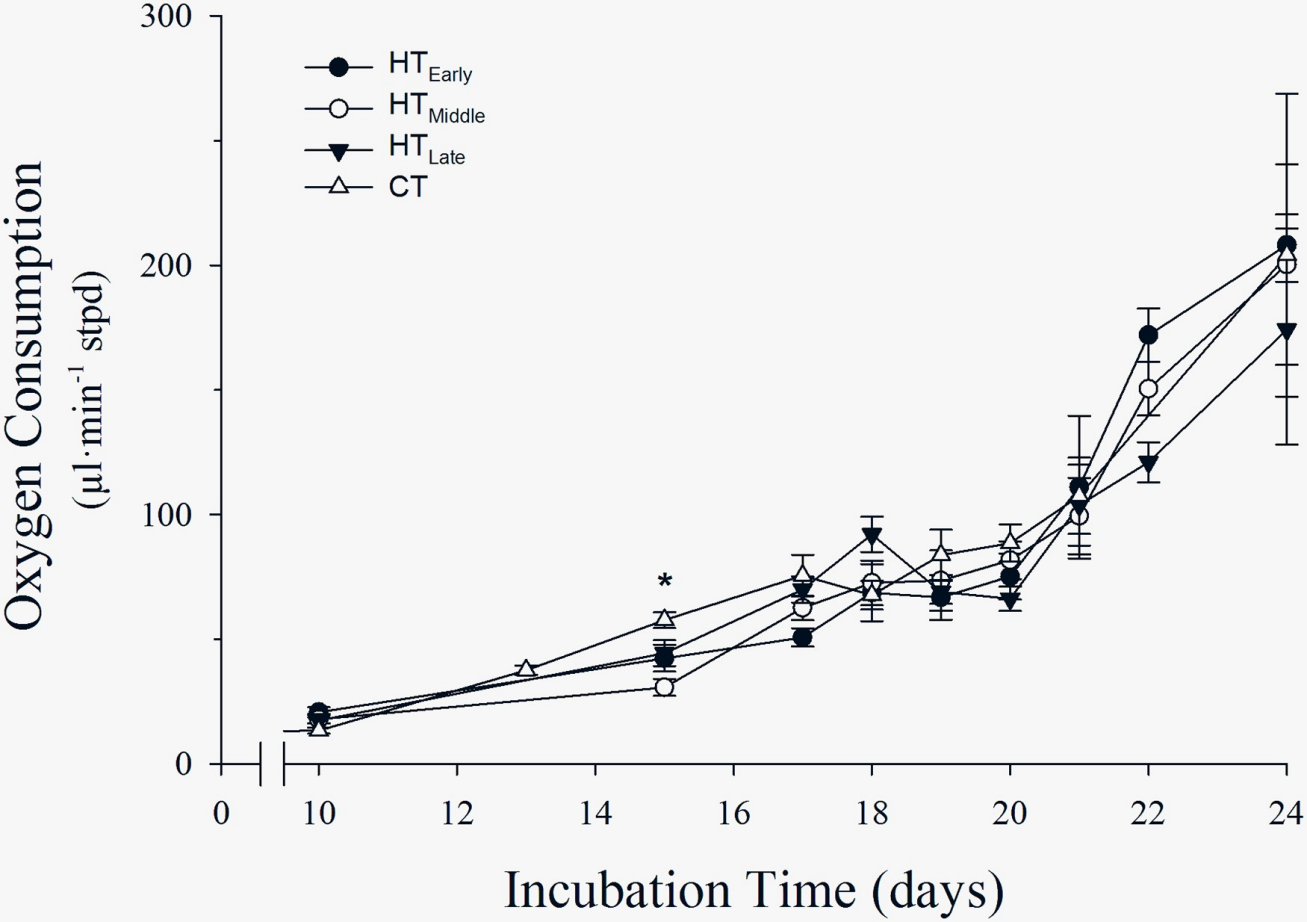
Oxygen consumption (mean ± S.E.) of incubating northern bobwhite embryos following exposure to commercial temperatures (CT) and high temperatures (HT) in the early, middle, or late third of a 12-d pre-incubation period. Asterisk indicates statistical difference between CT and HT treatments. Error bars indicate standard error.

Oxygen consumption was similar in overall pattern throughout incubation between acute exposure eggs (HT_Early_, HT_Middle_, and HT_Late_; Fig 3). However, on day 15, Vo2 for CT eggs was higher than Vo2 for HT_Early_, HT_Middle_, and HT_Late_; which were equal. For all other time periods, oxygen consumption among groups was not different (Fig 3).

### Hatchling development

Mean hatchling wet mass was different among treatment groups experiencing different pre-incubation treatments (P = 0.009). CT hatchlings were the heaviest with a mean wet mass of 7.04 g ± 0.17. Treatment groups had significantly lower mean wet mass than CT eggs: HT_Early_ = 6.26 g ± 0.20, HT_Middle_ = 6.11 g ± 0.24, and HT_Late_ = 6.40 g ± 0.29, although not different. Hatchling 3^rd^ toe length and beak length were not different among treatment groups (P = 0.14 and P = 0.30 respectively).

### Hatching rates

**Fig 4:**
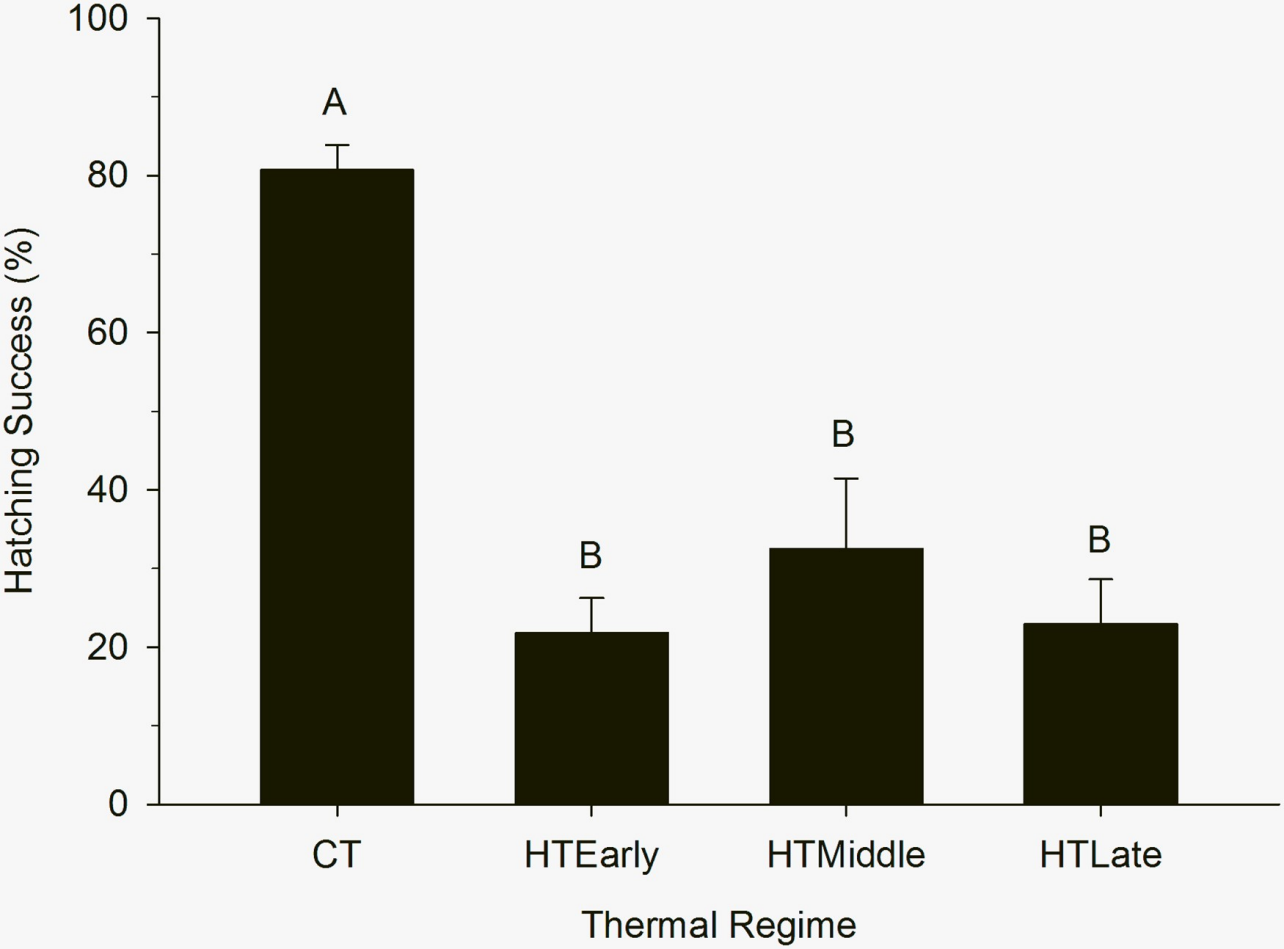
Mean hatching success of northern bobwhite eggs exposed to high temperatures (HT) in the early, middle, or late third of a 12-d pre-incubation period. Mean (± S.E.) shown, N = 30 eggs per group, letters indicate statistical groupings.

Mean hatch rates (± S.E.) for CT eggs (80.7% ± 3.1) were different from all treatment groups (Fig 4; P<0.001). Mean hatch rates were not significantly different among the treatment groups receiving acute doses of high heat (P = 0.48): HT_Early_ (21.7% ± 4.4), HT_Middle_ (32.5% ± 8.9), and HT_Late_ (22.9% ± 5.7).

**Fig 5:**
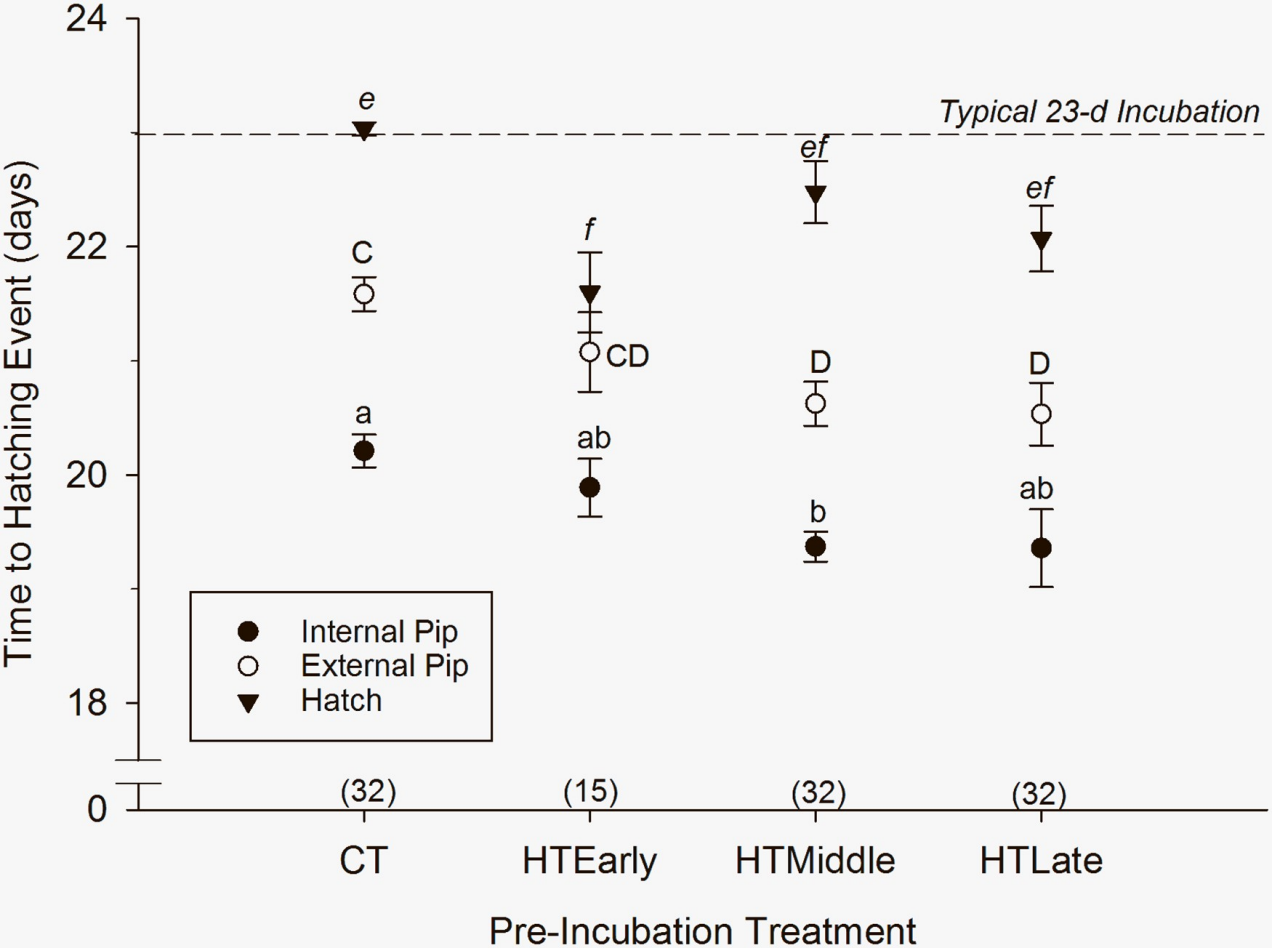
Time to internal and external pipping, and hatching for northern bobwhite eggs exposed to thermal treatments during a 12-d pre-incubation period. Data are presented as mean (± S.E.). Letters indicate statistical groupings, sample size in parentheses.

The mean time to internal pipping (i.e., the onset of pulmonary respiration) was affected by pre-incubation treatment with a significant difference between CT and HT_Middle_ treatment groups (Fig 5; P<0.001). The mean time to internally pip for CT eggs was 20.2 d ± 0.1, which was not different from HT_Early_ eggs (19.8 d ± 0.2), or HT_Late_ eggs (19.3 d ± 0.3), but was significantly higher than HT_Middle_ eggs (19.3 d ± 0.1; P = 0.014). Among the treatment groups, time to internal pip was not different (Fig 5; P = 0.06).

The mean (± S.E.) time to external pipping was significantly different between CT and some treatment groups (P<0.001), but not within HT groups (P = 0.996). CT eggs externally pipped at 21.5 d ± 0.1, equal to HT_Early_ eggs (21.0 d ± 0.3). HT_Middle_ eggs had a mean time to external pip of 20.6 d ± 0.1 which was not different than HT_Late_ eggs (20.5 d ± 0.2), or HT_Early_ eggs (Fig 5).

The mean (± S.E.) time to hatch was significantly different between CT (23.0 d ± 0.0) and HT_Early_ eggs (21.6 d ± 0.3; Fig 5; P<0.001). All other groupings were not different (P = 0.114). The mean (± S.E.) time to hatch for HT_Middle_ and HT_Late_ eggs was 22.4 d ± 0.2, and 22.0 d ± 0.2, respectively (Fig 5).

Mean (± S.E.) duration between internal pip and external pip was not different among groups (Fig 5; P<0.060). Mean (± S.E.) duration between external pip and hatch was significantly different among thermal groups (Fig 5; P<0.001). HT_Early_ eggs (0.5 d ± 0.0) exhibited the shortest duration between external pip and hatch (P<0.05). HT_Middle_ eggs (1.9 d ± 0.0) took longer than CT (1.5 d ± 0.1), HT_Early_ eggs but was not different than HT_Late_ eggs (1.5 d ± 0.0). The duration between internal pip and hatching was not different between CT (2.8 d ± 0.1), HT_Middle_ (3.1 d ± 0.1), and HT_Late_ (2.7 d ± 0.0). HT_Early_ eggs took the least amount of time to execute hatching (1.7 d ± 0; Fig 5).

### Hatching synchrony

Acute exposure groups (HT_Early_, HT_Middle_, and HT_Late_) received the same amount of heating degree-hours during pre-incubation; however, HT_Late_ is the only group that consistently exhibited hatching synchrony (all eggs hatched within 24 h). HT_Early_ eggs hatched across the range of 20–24 d, and HT_Middle_ eggs hatched across the range of 21–24 d. CT eggs hatched consistently at the standard incubation time of 23 d.

### Survival curves

**Fig 6:**
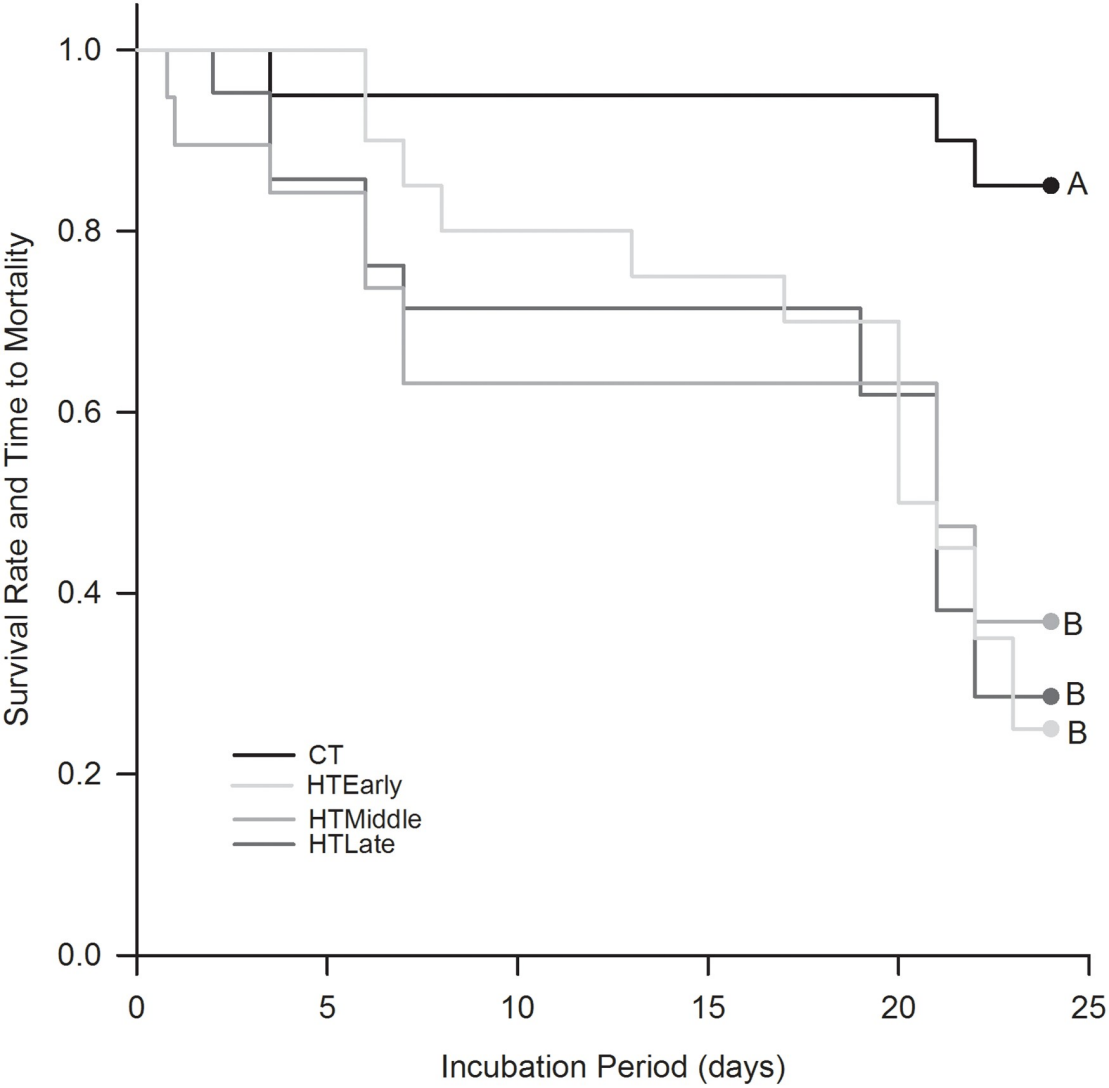
Kaplan-Meier survival curves showing survival rate and time to mortality of northern bobwhite embryos exposed to high temperatures (HT) in the early, middle, or late third of a 12-d pre-incubation period. Stair-step declines show time of mortality. N = 30 eggs per treatment. Letters indicate statistical groupings.

Mortality occurred in all groups prior to hatching. The survival rate and stage of development at the time of death was significantly different between CT and all HT groups (Fig 6; Log rank survival analysis, P<0.001). CT eggs had a mean survival time of 22.7 d ± 1.3 (P = 0.05) during the 23-d incubation. Although different from CT, survival time of HT_Early_ (18.4 d ± 1.5), HT_Middle_ (16.1 d ± 2.1), and HT_Late_ (17.1 d ± 1.8) were not different from each other (P = 0.98).

## Discussion

### Development

This study demonstrates that developmental plasticity occurs and critical periods of development exist in northern bobwhite embryos as a result of the timing and duration of exposure to equal and acute doses of hyperthermic oscillatory temperatures during pre-incubation. Development was more advanced in bobwhite embryos exposed to hyperthermic oscillating temperatures during the first 2/3 of pre-incubation as compared to eggs incubated in the last 1/3 of pre-incubation, which developed equally to eggs subjected to normal oscillating temperatures in [4]. Thus, bobwhites are more developmentally responsive in the first 2/3 of pre-incubation.

Although HT_Late_ embryos were less developed than HT_Early_ and HT_Middle_ eggs after pre-incubation, hatchling mass was not different among acute HT groups. This indicates compensatory growth of HT_Late_ embryos during incubation; however, a differential growth rate among treatment groups was not captured by Vo2 measurements during incubation. Further, CT embryos with no pre-incubation development, had a greater hatchling mass than all HT eggs, but no difference in Vo2 was detected between CT and HT groups except on day 15 of incubation.

Interestingly, CT eggs exposed to the commercial temperature and having no development during pre-incubation required much less heating degree-hours (6900 total heating degree-hours) than HT eggs in oscillating temperature groups (8488.8 total heating degree-hours) to advance to the same stage of development and larger hatchling mass. CT eggs only developed during incubation. This further illuminates that the nature of thermal exposure (i.e., constant versus oscillating), duration (i.e., chronic versus acute), and timing (e.g., different thirds of pre-incubation) of thermal exposure impact northern bobwhite development.

### Hatching

Acute 4-d exposure to hyperthermic oscillations during pre-incubation altered the timing of hatching, hatching events, and hatching synchrony. At least one of the HT exposed egg groups internally pipped, externally pipped, and hatched 0.5–1.4 d earlier than commercial temperature (CT) egg groups and the low temperature oscillation group (simulated non-drought) of Reyna and Burggren [4]. Further, the results are similar, although reduced in magnitude, to results of bobwhite eggs exposed to 12 d of high oscillating temperatures in [4], which internally pipped, externally pipped, and hatched 2 d earlier than low temperature oscillations and commercial temperatures. This indicates that both acute (4-d) and chronic (12-d) exposure to high oscillating temperatures induced an early onset of air breathing, external pipping, and hatching.

Accelerated hatch was likely the result of duration of exposure to high oscillating temperatures and associated development rather than just heating degree-hours. Eggs exposed to HT for 12 d in [4] had 2548.8 heating degree-hours, 2.2 d of pre-incubatory development and hatched 2.2 d early. Eggs exposed to HT for 4 d in the 1^st^ 2/3 of development had 1588.8 heating degree-hours, ~1 d of pre-incubatory development, and hatched ~1 d early. However, eggs exposed to HT for 4 d during the last 1/3 of pre-incubation had the same amount of heating degree-hours (1588.8), 0.5 d of pre-incubatory development yet hatched 1 d early. Eggs that were not exposed to hyperthermic oscillating temperatures did not hatch early. Eggs exposed to normal oscillating temperatures in [4] had 1108.8 heating-degree hours, 0.5 d of pre-incubatory development hatched at the same time (~23 d) as eggs exposed to commercial temps with no heating degree-hours or development during pre-incubation. This suggests that in addition to the nature and magnitude of thermal exposure, the duration (i.e., chronic versus acute) of thermal exposure may impact hatch timing of northern bobwhites. Further, groups receiving any duration of hyperthermic oscillations exhibited a reduced incubation period, which is perhaps an adaptation to the thermally stressful environments that northern bobwhites inhabit.

Hatching success and hatching synchrony were also affected by exposure to acute hyperthermic thermal stress. Hatching success appears to be inversely proportional to the quantity of heating-degree hours received during pre-incubation. Commercial eggs with no heating degree-hours in pre-incubation had the highest hatch success (80.7%), followed by eggs subjected to normal oscillating temperatures in [4] (53.4% hatch success, 1108.8 heating-degree hours). Eggs acutely exposed to hyperthermic oscillating temperatures for 4 d received 1588.8 heating degree-hours and had a hatching rate of 21–32.5%, and eggs with a chronic 12-d exposure in [4] received 3548.8 heating degree-hours and had a hatch rate of 5.9%. Surprisingly, hatching synchrony only occurred in eggs exposed to hyperthermic oscillating temperatures in the last 1/3 of pre-incubation. Although the other treatment groups received the same amount of thermal stress, early exposure in the first 2/3 of pre-incubation resulted in a loss of hatching synchrony. This is likely a result of the varying thermal regimens during the first 2/3 of pre-incubation instead of solely the result of hyperthermic exposure. Reyna and Burggren [4] demonstrated hatching synchrony in eggs exposed to 12 d of hyperthermic oscillating temperatures (simulated drought) and normal oscillating temperatures (simulated non-drought).

### Survival

One factor contributing to reduced hatching success was reduced survival during incubation when exposed to hyperthermic oscillating temperatures during pre-incubation; this was noted by the time to mortality during the 23-d incubation. Eggs exposed to 4-d of hyperthermic oscillating temperatures had reduced survival (16–18.4 d) as compared to commercial eggs (22.7 d). Reyna and Burggren [4] found eggs exposed to 12 d of normal and hyperthermic oscillating temperature had a mean survival time of 22.1 d and 12.2 d respectively, during the same time period. Thus, acute (4-d) and chronic (12-d) exposure to hyperthermic oscillating temperatures reduces survival during incubation.

### Implications

One major finding of this study was determining that acute 4-d doses of hyperthermic oscillating temperatures have the same or similar negative effects as chronic 12-d exposure during the pre-incubation period [4]. Similar to chronic exposure, acute exposure resulted in rapid pre-incubation development and reduced incubation time in what appears to be adaptations to thermal extremes across the northern bobwhite’s natural range. However, the consequences of acute egg exposure to hyperthermic oscillating temperatures are severe. The data revealed a 39–59% reduction in hatching success for eggs exposed to acute hyperthermic oscillating temperatures as compared to eggs exposed to normal oscillating temperatures [4]. For comparison, chronic exposure to hyperthermic temperatures reduced hatching by 88.96% as compare to normal oscillating temperatures [4]. Further, acute exposure in this study during the first 2/3 of pre-incubation resulted in hatching asynchrony, which likely increases predation for hatchlings and hens. These data are solid evidence that acute and chronic hyperthermic nest temperatures during the pre-incubation period could result in the observed reductions in the percentage of juveniles in natural populations during hot and droughty years [26, 27]. It is important to know that a few hot days during pre-incubation have an impact similar to longer periods of heat, especially with population variability around weather in the semi-arid regions of the bobwhite’s range [19, 26, 27]. These data could be useful to better model population success in a breeding season or to predict seasonal population success ahead of hunting season based on weather info. If nothing else, this study demonstrates the severe consequences encountered by hens who produce nests in extreme climates. The results could have cascading effects, particularly since bobwhites are a bird with a high socio-economic impact [35] and are an ecological indicator of the health of grasslands and grassland birds [36].
